# Case report and literature review: An intrahepatic sewing needle in a child

**DOI:** 10.3389/fped.2022.1101163

**Published:** 2023-01-09

**Authors:** Hao Shi, Zhibao Lv, Weijue Xu, Qingfeng Sheng, Xiong Huang, Ting Xu

**Affiliations:** Department of General Surgery, Shanghai Children's Hospital, School of Medicine, Shanghai Jiao Tong University, Shanghai, China

**Keywords:** intrahepatic, foreign body, children, needle, surgery

## Abstract

**Background:**

The presence of intrahepatic foreign bodies is a rare occurrence at the emergency department. Normally, foreign bodies reach the liver through migration. Incidence is lower among children than among adults, and the circumstances of children are often different. We report a 19-month-old boy with a sewing needle in the liver and review the previous reports of intrahepatic sewing needle in the PubMed database from the last three decades.

**Case presentation:**

A 19-month-old boy was transferred to our center from a local hospital presenting intermittent cough and rhinorrhea. A chest radiograph to exclude pulmonary disease revealed an incidental finding of a high-density shadow in the hepatic region. On admission, the boy had no gastrointestinal symptoms. Abdominal physical examinations were unremarkable. His mother, a worker in a textile factory, denied any history of trauma. Abuse was excluded based on investigation. Preoperative routine test results were normal. Contrast-enhanced computed tomography (CT) revealed that the sewing needle was located in hepatic segment IV and the tip had close relationship with intrahepatic portal vein. Initially, laparoscopy was performed without success. We eventually converted to laparotomy to completely remove the rusty sewing needle. The patient resumed feeding soon after the operation and was discharged in a few days.

**Conclusions:**

Intrahepatic sewing needle has high incidence among boys and developing countries. Combined with contrast-enhanced CT, knowledge of the pediatric patient's family background and medical history would help judge the route of entry and determine the management and surgical strategy. Laparoscopic procedure is not suitable for rusty sewing needles.

## Background

The presence of foreign bodies *in vivo* is a worldwide problem among adults and children. Mostly, foreign bodies enter the body through natural orifices and pass spontaneously without causing complications (1). Due to the lack of communication channels between the internal and external environments, foreign bodies rarely appear in solid organs (2–4). In clinical practice, most cases of intrahepatic foreign bodies occur in children, elderly individuals, psychiatric patients, and alcoholic population (5). Retrieved foreign bodies from the liver including sewing needles, hair pins, military equipment, and some foreign bodies may be iatrogenic (6). Various routes of migration of foreign bodies include swallowing, percutaneous route, iatrogenic, and hematogenous translocation (7). Few pediatric cases of intrahepatic sewing needle have been reported in the past. The patients in these studies have differences in clinical manifestations, location, route to liver, and management. Here, we report a case of a 19-month-old boy who was eventually diagnosed with a sewing needle in the liver, and we review the literature related to intrahepatic sewing needle among pediatric patients in the PubMed database in the past 30 years.

## Case presentation

**Figure 1:**
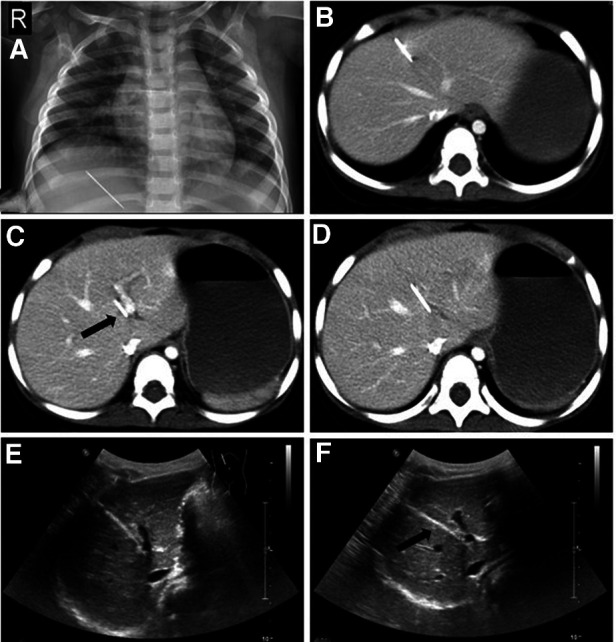
Preoperative imaging examinations of the patient. (**A**) An accidental chest x-ray showing a striped high-density shadow in the hepatic region. (**B–D**) Contrast-enhanced CT showing the major part of the sewing needle trapped in the liver parenchyma with the tip closest to the sagittal part of the left branch of the portal vein. (**E,F**) Ultrasound images showing a minimal distance between the sewing needle and portal vein.

A 19-month-old boy presented to the local hospital with upper respiratory symptoms, such as cough and rhinorrhea. A chest x-ray was performed to evaluate the lungs and exclude pneumonia. Thereafter, a striped high-density shadow was recognized in the hepatic region by chance. The shadow with a line hole at the end of one side suggested the presence of a sewing needle (Figure 1A). Two days later, the child was transferred and admitted at our department. He had no complaints of discomfort. Aside from the upper respiratory tract infection symptoms, he did not present with any gastrointestinal symptoms, such as abdominal pain, vomiting, and distension, and systemic symptoms, such as fever. On examination, the patient was fully conscious and responsive. A specialized abdominal physical examination was unremarkable without hepatic region tenderness, rigidity or guarding, jaundice, or any palpable abdominal mass. No wound healing manifestations, such as scarring and hyperpigmentation, were found. The mother, an embroiderer in a textile factory, denied a history of trauma. Based on the witness of neighbors and relatives, we excluded the possibility of parental abuse.

Routine blood test, coagulation test, blood chemistry, and other preoperative routine laboratory examinations were performed. Major indicators, including leukocyte count, hemoglobin (Hb), hematocrit (Hct), alanine aminotransferase (ALT), and aspartate aminotransferase (AST), were within the normal range. The patient's C-reactive protein (CRP) level was 8 mg/L (≤5 mg/L); the mild elevation was associated with upper respiratory tract infection. To identify the accurate location of the foreign body, contrast-enhanced computed tomography (CT) scans and abdominal ultrasound were performed. The imaging confirmed the sewing needle (approximately 3.7 cm long) to be present in the liver and located in hepatic segment IV. Unexpectedly, the sewing needle seemed to have entirely entered the liver with the tip adjacent to the sagittal part of the left branch of the portal vein (Figures 1B–F). Combined with the clinical manifestations, laboratory examinations, and imaging findings, we believed that the tip had not injured the intrahepatic portal vein. However, as a growing boy with increasing physical activity, the patient was at a high risk of hemorrhage.

**Figure 2:**
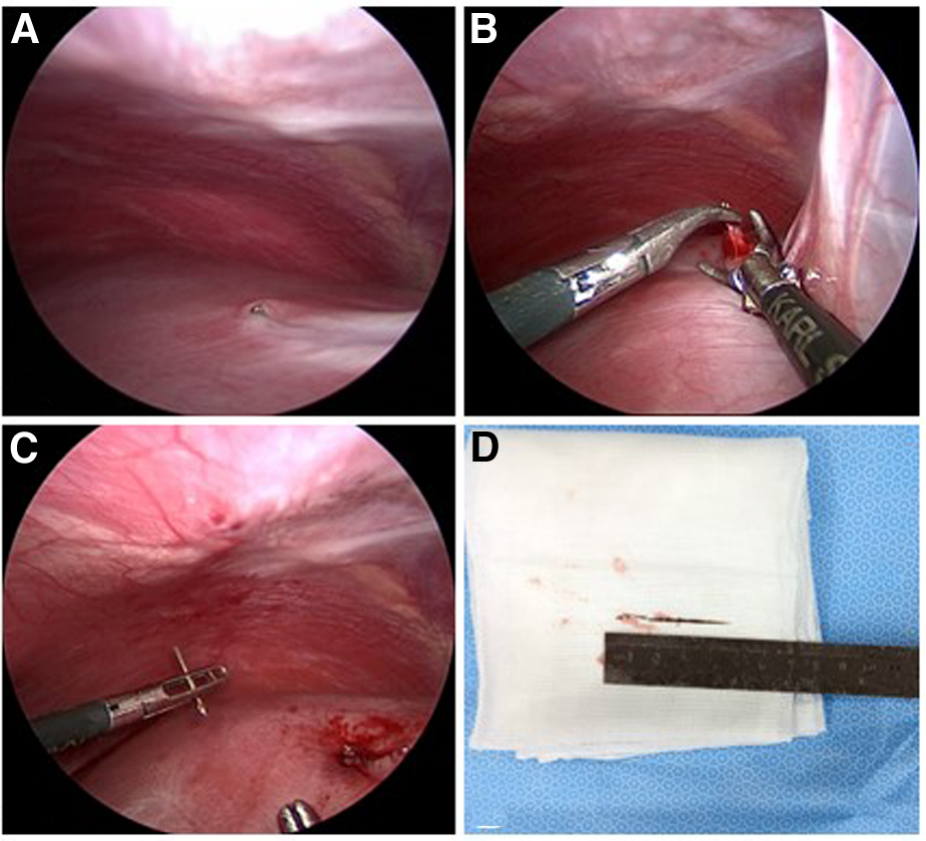
Intraoperative findings. (**A**) The line hole of the sewing needle exposed the surface of the liver and was partially covered by hepatic capsule. (**B,C**) The sewing needle broke into pieces after clamping and pulling. (**D**) The lower and middle parts of the sewing needle were rusty; and the length was 3.7 cm.

Laparoscopy was chosen as the initial approach for surgery. Intraoperative findings revealed that the enlarged part of the line hole was only exposed slightly above the surface of the liver (Figure 2A). No peritoneal scarring, adhesions, or bloody ascites were found. At first, we tried to remove the sewing needle by clamping and pulling (Figure 2B). However, the middle and lower parts of the sewing needle were tightly trapped in the liver parenchyma. Concerned about possible damage to the portal vein, we had to control the operation in a minimal range of motion. Due to the prolonged retention in the humid environment, the ferrous sewing needle had corroded and become brittle. As a result, the sewing needle broke into parts in the process of removal (Figure 2C). We eventually removed the sewing needle by converting to laparotomy and sutured the incision of the liver parenchyma. Rust had covered the lower and middle parts of the sewing needle (Figure 2D). By comparing the actual length of the needle with that measured on imaging, we confirmed that the sewing needle had been completely removed. The child resumed normal diet 1 day after surgery and was discharged 3 days after the operation. No complications were reported during the postoperative 1-month follow-up period.

## Discussion

Preschool children are at the stage of accelerated development of intelligence, increased range of activities, and increased contact with external objects. This age group has the highest prevalence of foreign body ingestion due to low awareness of danger and ability of self-protection. In the United States, approximately 70,000 cases of foreign body ingestion are reported annually in children under 5 years of age (8). However, foreign bodies rarely retain in solid organs of pediatric patients. A systematic review of the literature on intrahepatic foreign bodies in the pediatric population suggested that only 16 English cases were reported in the last two decades (6). To our best knowledge, few pediatric cases of intrahepatic sewing needle have been reported in current literature (Table 1). Among the 12 patients, the mean age of the patients was 4.6 years (range: 0.3–16.0 years) with a male-to-female ratio of 3:1. Ten (83.3%) patients were below the age of 5 years. These findings correspond with the high incidence of foreign body ingestion based on gender and age. Interestingly, most cases (72.7%) were reported by researchers in developing countries, probably because the textile industry is an important industry of the economies of these third world countries, where manual manufacturing is widely adopted.

**Table 1:** Literature review of intrahepatic sewing needle in pediatric patients.

Authors	Year	Country	Case	Potential path	Sex	Age (years)	GI symptoms	Laboratory test indicators	Hepatic segment or lobe	Management	Length (cm)	Rusty or not	Prognosis
Crankson (9)	1997	Saudi Arabia	1	Unknown	M	2	None	NG	R	Observation	NR	NR	Good
Nishimoto et al. (10)	2003	Japan	1	Percutaneous	M	1	None	Normal	L	Laparotomy	4.0	NR	Good
Le Mandat-Schultz et al. (11)	2003	France	1	Unknown	M	0.9	None	Normal	R	Laparoscopy	2.4	NR	Good
Azili et al. (12)	2007	Turkey	1	Swallow	F	14	Abdominal pain	Increased WBC with left shift Increased levels of ESR	VI	Laparotomy	NR	NR	Good
Dominguez et al. (13)	2009	Switzerland	1	Unknown	M	3	None	Normal	I	Laparoscopy	NR	Yes	Good
Saitua et al. (14)	2009	Chile	1	Percutaneous	M	0.3	None	Normal	IV	Laparotomy	6.0	NR	Good
Avcu et al. (15)	2009	Turkey	1	Swallow	F	16	Abdominal pain Nausea Vomiting	Increased WBC with left shift Increased levels of ESR, CRP, AST, ALT	V	Antibiotic therapy + Laparotomy	3.0	NR	Good
Akçam et al. (16)	2009	Turkey	1	Swallow	M	5	None	Normal	R	Endoscopy	3.8	Yes	Good
Xu et al. (17)	2013	China	1	Unknown	M	5	None	Increased WBC	R	Laparotomy	5.0	Yes	Good
Xing et al. (5)	2022	China	2	Unknown	F	4.7	None	Normal	IV, V, VI, VII	Laparotomy	4.0	Yes	Good
				Percutaneous	M	2	Abdominal pain	Normal	VI	Laparotomy	3.5	No	Good
Our case	2022	China	1	Percutaneous	M	1.6	None	Normal	IV	Laparoscopy + laparotomy	3.7	Yes	Good

ALT, alanine aminotransferase; AST, aspartate aminotransferase; CRP, C-reaction protein; ESR, erythrocyte sedimentation rate; GI, gastrointestinal; L, left; NG, not given; NR, not recorded; R, right; WBC, white blood cell.

The occurrence mechanism of foreign bodies in solid organs is complicated because of the lack of connection channels between solid organs and external environment. Intrahepatic foreign bodies are usually caused by secondary injuries since the liver locate in the abdominal cavity and covered by ribs. Several potential routes for foreign bodies entering the liver have been reported: (1) Migration through the gastrointestinal tract after ingestion which is the most frequent route; (2) puncture through the abdominal or thoracic wall; (3) hematogenous spread; (4) iatrogenic causes associated with severe medical errors (7, 13). In adult cases, the most common intrahepatic foreign body is fishbone. Other bodies, such as toothpick, hairpin, round, bullet, pen, pin, and dental plate have been previously reported (18). Most intrahepatic foreign bodies in adults derive from the gastrointestinal tract (19). The left lobe of the liver is commonly affected because the stomach and duodenum are the most common sites of perforation by foreign bodies (20). The right lobe of the liver is rarely affected by migration of foreign bodies from the ascending colon. Due to intestinal bacterial translocations, associated chronic diseases, and immune deficiency of the elderly, liver abscess is a common complication in adult patients with related symptoms, including fever, chills, and abdominal pain (4, 21). Nevertheless, the situation seems to be different in children. Except for migration through the gastrointestinal tract, the incidence of foreign bodies entering the liver by other routes is much higher. The occurrence has close relationship with children's accidental trauma or abuse. Due to restricted ability of language expression, crying is the only way for younger children to express physical discomfort, which is often misunderstood by guardians as hunger, emotional changes, and urination or defecation. To our best knowledge, sewing needle is the most common intrahepatic foreign body in children. Migration through the gastrointestinal tract and percutaneous puncture are the major routes. Complications, such as liver abscess, hepatic granuloma, and inflammatory pseudotumor, were rarely reported in pediatric patients. This may relate to dietary heterogeneity and different colonization of the intestinal flora. In our study, the sewing needle was identified by incidental radiographic examinations, as the patient did not present with any gastrointestinal or systemic symptoms. In previous reports, only three patients presented with abdominal pain and all other patients were asymptomatic. Two of these patients swallowed the sewing needle and presented with gastrointestinal symptoms with elevation of inflammatory indicators.

The patient's history is important in finding the clue for making the proper diagnosis. However, a specific medical history with the intention to determine the timing and modality of ingestion or trauma is challenging especially for older and younger patients (6). Moreover, the social affiliations including occupation living conditions, and witness from neighbors were necessary. Precise radiological imaging is essential to tailor the operative pathways and protocol. Contrast-enhanced CT is a sensitive method for diagnosing foreign bodies especially in coronal or sagittal reconstruction. Compared with abdominal radiography, CT can not only find all foreign bodies but also mark the position, shape, and adjacent relationship with surrounding organs (21, 22). The orientation of the tip of the sewing needle may suggest a possible route of entry. However, metals generate typically high- and low-density streak artifacts on CT, which makes the identification of the relationship between the needle and vessels challenging. Typical metallic artifacts in coronal reconstruction were observed in our case. To solve this problem, an expert performed abdominal ultrasound.

Retention of foreign bodies in the liver is a rare occurrence. Therefore, management remains controversial, and treatment strategy relies on the estimated risk–benefit ratio of conservative management and intervention, personal experience, and sound judgment (13). Crankson (9) performed an observation strategy for a 2-year-old boy with intrahepatic needle in the right robe. A regular 3-year follow-up confirmed that the boy remained asymptomatic, and the position of the needle had not changed. Leading to a heavy burden in terms of immediate and long-term complications, prophylactic extraction was recommended at several centers (17). Although the patient in our case was completely asymptomatic, a surgical procedure was performed for several reasons. First, considering that the tip of the sewing needle was extremely close to the portal vein, it had a great possibility of shifting with body movement to cause vascular injury. Second, the patient may undergo evaluation for hepatic conditions for other diseases in the future, and the metal artifact would affect imaging. Third, the persistence of foreign bodies inside the hepatic parenchyma may cause severe and long-term complications. Endoscopy is a well-established technique for removing parts of foreign bodies in the intestinal tract. However, after foreign body migration and mucosal healing, foreign body may be difficult to find (20). Laparoscopic surgery has multiple advantages, namely mild postoperative pain, short hospitalization time, and cosmetic benefits (7). Laparoscopy before laparotomy is recommended to confirm the need for open abdominal access (23). Some needle would completely trap in liver parenchyma over time that could not be visualized during laparoscopy. A conversion to laparotomy combined with intraoperative ultrasound is recommended to localize the needle and detect the relationship between the vessels and needle, which is a great guidance to the operator. In our case, laparoscopy was performed as the primary surgical strategy. A rusted sewing needle was found due to the long-term exposure to a humid and warm environment. To achieve minimal invasiveness, as the choice of the patients, we tried to remove the needle with laparoscopic instruments. However, the sewing needle broke into parts and had to convert to laparotomy to complete the surgery. Our experience showed laparoscopy to be helpful in confirming the sewing needle's location and surrounding conditions of adhesion and edema. However, in cases where physical properties have been altered, laparoscopy-guided removal is inappropriate. Additionally, minimal invasiveness is not needed when complete removal of foreign bodies is required.

## Conclusion

Intrahepatic sewing needles are rare occurrences in children. Contrast-enhanced CT and history-taking helped locate the needle, suggest a possible route of entry, and provide a reference for the operative approach. To prevent potential complications, removal of the sewing needle should be considered even if patient is asymptomatic.

## Data Availability

The raw data supporting the conclusions of this article will be made available by the authors, without undue reservation.
